# Accessory Spleen: A Rare and Incidental Finding in the Stomach Wall

**DOI:** 10.7759/cureus.24977

**Published:** 2022-05-13

**Authors:** Sophie G Trujillo, Sherif Saleh, Ryan Burkholder, Fahmi Shibli, Bhavesh Shah

**Affiliations:** 1 Internal Medicine, MetroHealth Medical Center, Cleveland, USA; 2 Gastroenterology and Hepatology, MetroHealth Medical Center, Cleveland, USA

**Keywords:** gastro-intestinal neoplasm, endoscopic ultrasound (eus), ultrasound-guided, endoscopy, accessory spleen

## Abstract

An accessory spleen is splenic tissue located separately from the anatomical location of the spleen and is a rare phenomenon. It can be found within the gastrointestinal tract. Clinically, accessory spleens are benign but can be misidentified as reactive lymph nodes or malignant gastrointestinal tumors. They are often diagnosed via endoscopy or imaging. We report the case of a woman presenting with iron deficiency anemia who was incidentally noted to have a gastric submucosal lesion with pathology significant for accessory spleen. As this case illustrates, when submucosal lesions are present in the stomach, especially in patients with a history of splenectomy, the endoscopic ultrasound (EUS) operator should consider the possible presence of an accessory spleen to minimize invasive removal procedures.

## Introduction

The spleen lies in the left upper quadrant of the peritoneal cavity and is a vascular organ [1]. The spleen functions to maintain the homeostasis of the immune and hematologic systems [1]. An accessory spleen occurs when splenic tissue is in a separate body region, which occurs in up to 15% of the population [1]. The most common locations for accessory spleens are in the splenic hilum (80%) and the pancreatic tail (17%) [2-5]. However, they can also be located within the greater omentum and gastrointestinal tract [2]. They are usually benign and discovered as incidental findings through endoscopy, computed tomography (CT), or magnetic resonance imaging (MRI) [3,6].

Accessory spleens can arise from a congenital etiology or via splenosis [2,3]. The congenital etiology occurs during the fifth week of fetal development from a failed fusion of the splenic anlage [7]. Splenosis is the process of autologous transplantation of the spleen after splenectomy [2]. It can present as numerous nodules and have compensatory hypertrophy reaching up to 5 cm [3,4]. Unlike splenosis nodules, accessory spleens via congenital etiology have a capsule containing smooth muscle and elastic elements [3]. Splenosis nodules lack a hilus with blood vessels, and blood is supplied by accessory arteries that penetrate the nodule [3]. This case describes a unique presentation of an accessory spleen presenting as a submucosal gastric lesion. This article was previously presented as a meeting abstract at the American College of Gastroenterology Conference on October 25, 2021.

## Case presentation

A 54-year-old woman presented to her primary care physician for fatigue and was found to have iron deficiency anemia. Her past medical history was notable for stage 2 right breast cancer at age 24 (treated with right mastectomy, adjuvant chemotherapy, and tamoxifen), a splenectomy in her 30s for a large vascular mass, and a total abdominal hysterectomy at age 50 due to menorrhagia. Her family history was notable for her sister's death at age 26 due to splenic rupture of a vascular mass but was otherwise noncontributory. Immunization records were significant for pneumococcal, meningococcal, and Haemophilus influenza vaccinations two weeks prior to elective splenectomy. Her social history was negative for tobacco, alcohol, or drug use. Her laboratory evaluation revealed low hemoglobin, low mean corpuscular volume, low iron, low iron saturation, high total iron-binding capacity, high transferrin, and low ferritin (Table 1).

**Table 1:** Laboratory evaluations g/dL: grams/deciliter; fL: femtoliters; µg/dL: micrograms/deciliter; µg/mL: micrograms/milliliter; mg/dL: milligram/deciliter.

Analyte	Laboratory value	Reference range
Hemoglobin	10 g/dL	12−15 g/dL
Mean corpuscular volume	73 fL	80−100 fL
Iron	14 µg/dL	45−160 µg/dL
Iron saturation	3%	20−55%
Total iron binding capacity	535 µg/mL	250−410 µg/mL
Transferrin	382 mg/dL	210−375 mg/dL
Ferritin	4.1 µg/mL	10−219 µg/mL

**Figure 1:**
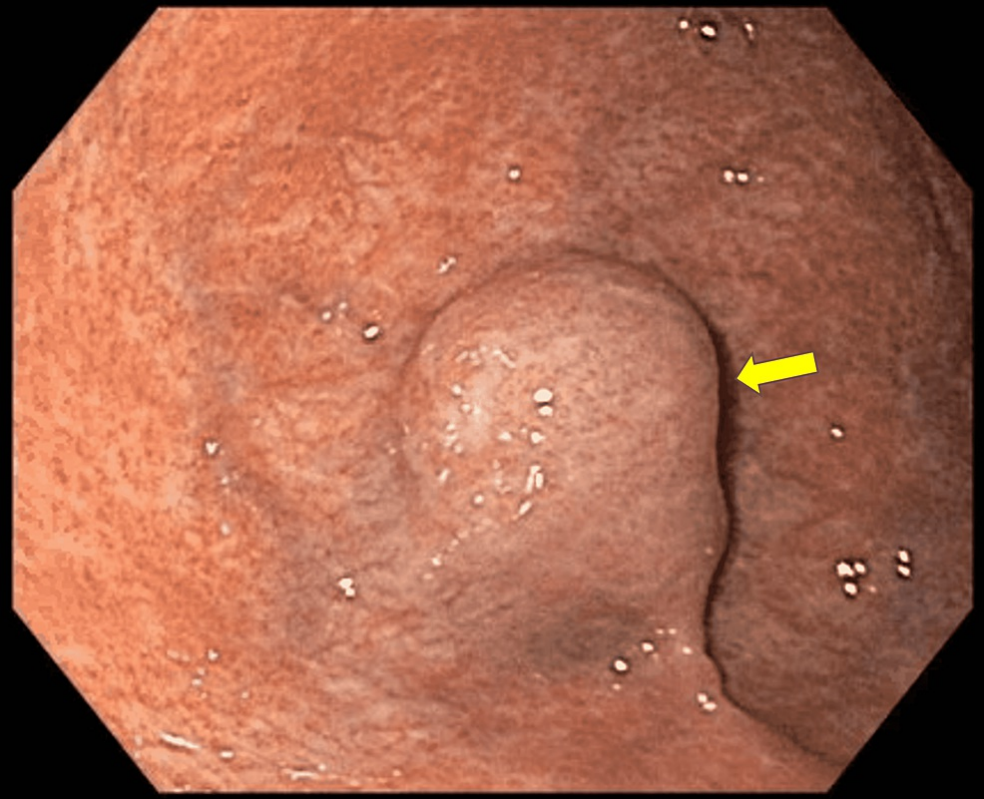
A 2 cm subepithelial gastric fundic lesion seen on EGD

**Figure 2:**
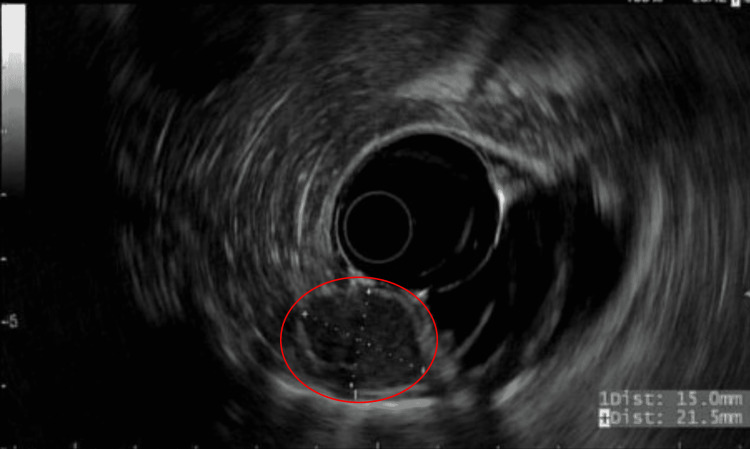
Ultrasound image with hypoechoic lesion measuring 1.5 cm × 2 cm

**Figure 3:**
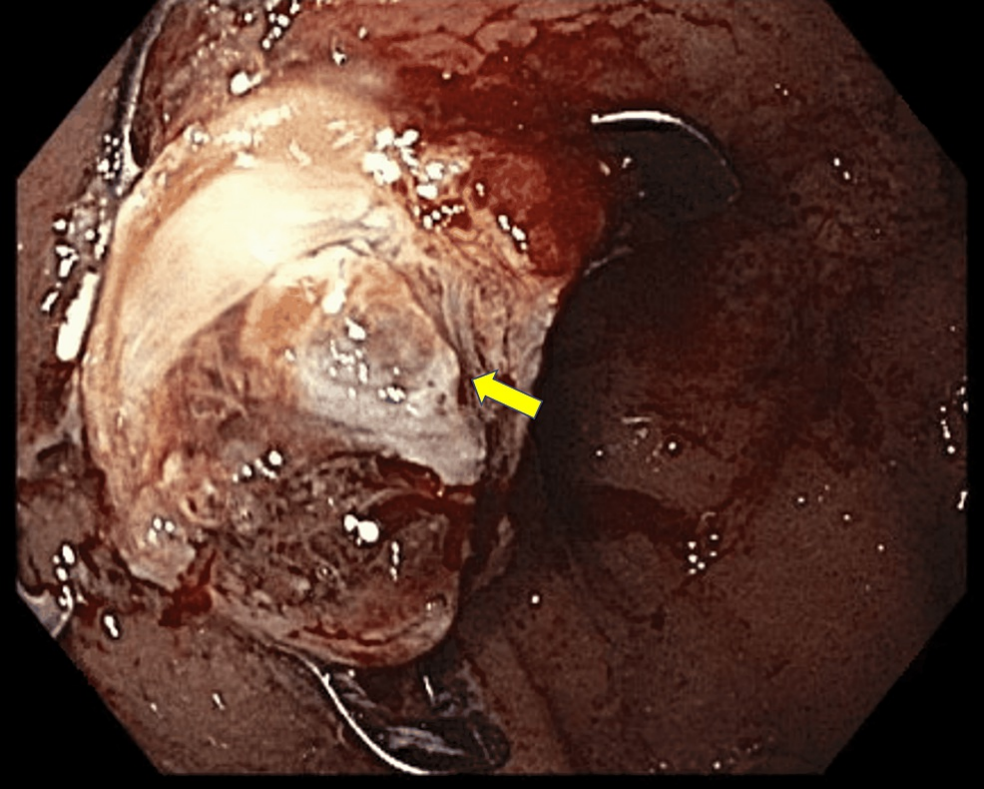
Full thickness resection of lesion with the FTRD® system

**Figure 4:**
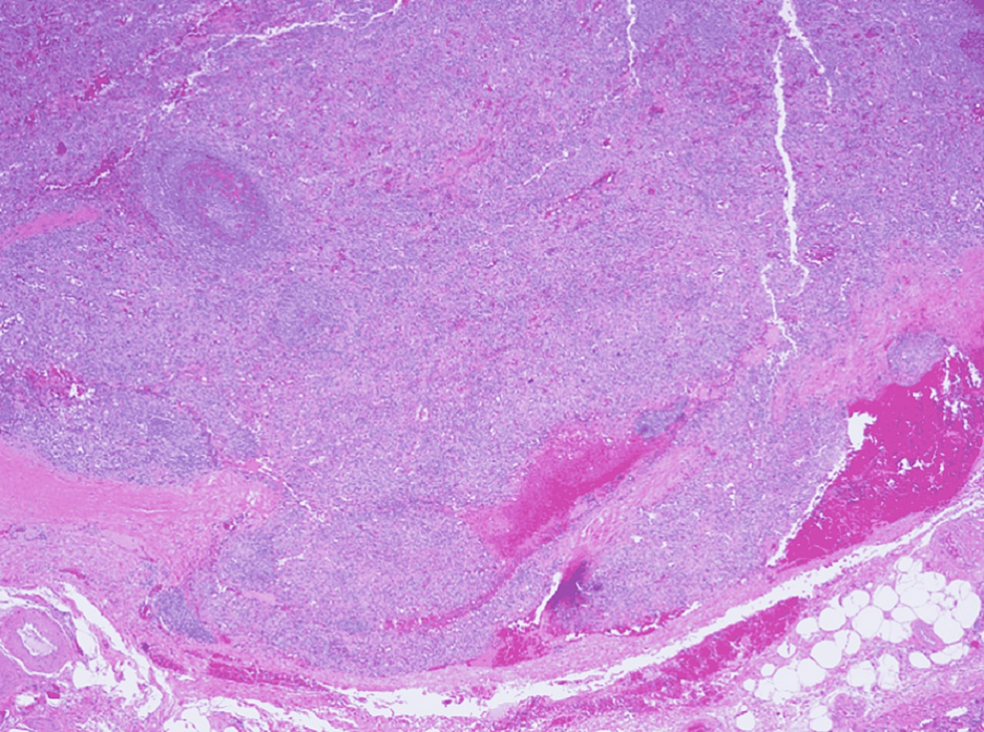
Pathology of submucosa with accessory spleen

As part of her anemia evaluation, the patient underwent an esophagogastroduodenoscopy, significant for a 2-cm submucosal gastric fundic lesion (Figure 1). Follow-up endoscopic ultrasound (EUS) for the gastric lesion revealed a homogenous, oval-like lesion, measuring 1.5 cm × 2 cm (Figure 2). We conducted a full-thickness resection of the lesion via the Ovesco Gastroduodenal FTRD (full-thickness resection device) System (Ovesco Endoscopy AG, Tübingen, Germany), and the patient had no complications (Figure 3). The pathology results were significant for the submucosal accessory spleen extending into the deep tissue end (Figure 4). Her anemia did not improve the following resection, and the gastric lesion was believed to be truly incidental and unrelated to her anemia.

## Discussion

Accessory spleens are additional splenic tissue located away from the anatomical site of the spleen and can have immunologic function [6]. There are a few case reports describing accessory spleens. Intragastric spleens are particularly rare [2]. Patients with gastric accessory spleens are commonly asymptomatic but may have associated upper abdominal pain [4]. Rare complications of the accessory spleen include spontaneous rupture, embolization, hemorrhage, and torsion [7].

Accessory spleens are most commonly an incidental finding on imaging or endoscopy. They are often misdiagnosed and mistaken for malignant submucosal tumors [2]. The CT and MRI imaging findings of an accessory spleen are usually small round masses (<2 cm) that enhance homogenously with attenuation identical to that of normal splenic parenchyma [4,6]. EUS is the best modality for viewing submucosal tumors. On EUS, the accessory spleen is rounded, and its echogenicity is identical to that of the spleen [4]. Technetium 99 m-sulfur colloid scintigraphy is a sensitive and specific imaging modality to detect splenic tissue and may assist in diagnosing an accessory spleen [7-9]. Given our patient's previous history of splenectomy, it was impossible to compare the accessory spleen's parenchyma to her spleen. The etiology of the accessory spleen is unknown but likely due to an autologous transplant of the spleen from her previous splenectomy.

Since accessory spleens are usually asymptomatic and of no clinical significance, resection is not recommended [2]. Compensatory hyperplasia of the spleen can occur after splenectomy, and resection may result in hematological diseases [2,7]. However, in cases where splenectomy was performed due to refractory immune thrombocytopenia, removing the accessory spleen may be indicated to prevent relapse [10]. Resection may also be indicated if the patient becomes symptomatic or if the tumor is indistinguishable from other tumors [2,7].

EUS-guided fine-needle aspiration may assist with the diagnosis of accessory spleens in cases of uncertainty to avoid invasive resections [7]. The cytologic features include clusters of lymphoid cells, large platelet aggregates, and prominent vasculature [11]. Additionally, CD8 immunostaining can be used for sinusoidal structures within a lymphoid aggregate, which is pathognomonic for splenic tissue [11]. Another cytopathologic finding that was seen post-splenectomy was a lack of Howell-Jowell bodies. Howell-Jowell bodies are nuclear DNA remnants of erythrocyte precursor cells that are typically phagocytosed by the normal spleen. Thus, in patients who are completely asplenic (whether functional or surgical), Howell-Jowell's bodies are typically undetectable. However, studies have suggested that the detection of Howell-Jolly bodies does not reflect splenic function accurately compared to determining the percentage of pitted erythrocytes for assessing splenic function [12].

## Conclusions

Accessory spleens are a rare occurrence and may have an immunological function. They are usually incidental findings with no clinical significance. However, they can resemble lymphadenopathy or malignant tumors and should be a differential diagnosis. When submucosal lesions are present in the stomach, especially in patients with a history of splenectomy, the EUS operator should consider the possible presence of an accessory spleen to minimize invasive removal procedures.
